# Identifying Protein Biomarkers in Blood for Alzheimer's Disease

**DOI:** 10.3389/fcell.2020.00472

**Published:** 2020-06-18

**Authors:** Tianyi Zhao, Yang Hu, Tianyi Zang, Yadong Wang

**Affiliations:** School of Life Science and Technology, Department of Computer Science and Technology, Harbin Institute of Technology, Harbin, China

**Keywords:** Alzheimer's disease, similarity of diseases, protein, minimum angle regression, elastic network

## Abstract

**Background:** At present, the main diagnostic methods for Alzheimer's disease (AD) are positron emission tomography (PET) scanning of the brain and analysis of cerebrospinal fluid (CSF) sample, but these methods are expensive and harmful to patients. Recently, more researchers focus on diagnosing AD by detecting biomarkers in blood, which is a cheaper and harmless way. Therefore, identifying AD-related proteins in blood can help treatment and diagnosis.

**Methods:** We proposed a hypothesis that similar diseases share similar proteins. Diseases with similar symptoms are caused by abnormalities of similar proteins. Assuming that the similarities between AD and other diseases obey the normal distribution, we developed an iterative method based on disease similarity (IBDS). We combined Elastic Network (EN) with Minimum angle regression (MAR) to find the optimal solution. Finally, we used case studies and Summary data Mendelian Random (SMR) to verify our method.

**Results:** We selected 39 diseases which are highly related to AD. They correspond 1,481 kinds of proteins. One hundred and eighty-four proteins are reported to be related to AD in Uniprot and the number would be 284 with our method. The AUC of our method by cross-validation is 0.9251 which is much higher than previous methods.

**Conclusion:** In this paper, we presented a novel method for prioritizing AD-related proteins. Seven proteins have tissue specificity in blood among these 284 proteins, which could be used to diagnose AD in future. Case studies and SMR have been used to prove the relationship between these 7 proteins and AD.

**Availability and Implementation:** https://github.com/zty2009/Identifying-Protein-Biomarkers-in-Blood-for-Alzheimer-s-Disease

## Introduction

Alzheimer's disease (AD) is a progressive neurodegenerative disease with insidious onset (Peng and Zhao, 2020). Clinical manifestations of AD include memory impairment, aphasia, agnosia, executive dysfunction, and personality and behavioral changes. At present, the only reliable diagnostic methods for AD are positron emission tomography and cerebrospinal fluid sampling and analysis through lumbar puncture.

Recently, finding alternatives to diagnosing AD has become a hot issue (Henriksen et al., 2014; Wang et al., 2018; Ren et al., 2019; Sun et al., 2019). Ray et al. found 18 plasma proteins have high specificity in AD patients. They then found that these proteins were associated with Aβ and tau levels in CSF. Then the Human Discovery Multi-Analyte Profile (MAP) has become a popular tool to identify plasma analytes. But, these exciting results raise a major issue that it is hard to reproduce these protein panels (Henriksen et al., 2014). Zetterberg et al. (2013) found that the correlation between CSF and plasma NFL was stronger than tau. Olsson et al. (2016) confirmed this view, and they found that the NFL was increasing in both AD patients and MCI's CSF. Studies have found this phenomenon in serum and plasma samples as well (Bacioglu et al., 2016). O'Bryant et al. (2014) used a serum-based algorithm to distinguish AD from Parkinson's disease and cross-validated this algorithm. Preische et al. (2019) found that the rate of change of serum neurofilament light chain (NFL) can be used to distinguish mutation carriers from non-mutation carriers. Westwood et al. (2016) used 12 years period data to find that seven plasma proteins are significantly associated with amyloid burden. Burnham et al. (2014) used a larger dataset and found nine- analyte signature. Rembach et al. (2014) measured Aβ_1−40_, Aβ_1−42_, Aβ_n−40_, and Aβ_n−42_ at baseline for 18 months. They found that Aβ_1−40_/Aβ_1−42_ was decreasing and inversely correlated with neocortical. Most recently, Akinori Nakamura et al. claimed that they can use amyloid-β precursor protein (APP) _669−−711_/Aβ_1−42_ and Aβ_1−40_/Aβ_1−42_ ratios, and their composites, to predict individual brain amyloid-β-positive or -negative status. However, Lövheim et al. (2017) found an opposite phenomenon that Plasma concentrations of free Aβ did not differ between preclinical AD cases. They sampled 339 preclinical AD cases and 339 age- and sex- matched dementia-free controls and used Luminex xMAP technology to determine concentrations of free plasma Aβ.

These paradoxical experimental results reflect the unreproducibility of the experimental results. A biological experiment always takes years and a large amount of money. Obviously, we can't just confine ourselves to Aβ, NFL, and tau. We need to find more AD-related proteins that can be abnormally expressed in the blood. Prioritizing AD-related proteins can help researchers save a lot of time and money (Zhao et al., 2019a).

At present, the most commonly used method to identify disease-related protein are protein-protein interaction network (PPI) and machine learning methods. Most PPI networks are built based on genes' relationship. Mukherjee et al. (2014) used dense module searching (DMS) method to integrate gene-wide association results into PPI network and identified candidate genes or sub-networks for AD. Lots of researchers used PPI to predict the function of protein and genes and identify the disease-related proteins and genes (Cheng, 2019; Cheng et al., 2019). In 2017, Li et al. (2017) developed a novel PPI network whose name is InBioMap. This network contains more interactions and better functional biological relevance. Although PPI network has excellent performance, it mainly has two drawbacks. Firstly, most studies of protein networks are based on static network models. However, static protein networks are highly average and idealized network structures. In fact, with the change of external conditions, some proteins will be degraded, while others will be transformed. This leads to disappearance of some protein interactions and the formation of new protein interactions (Li et al., 2019). The other drawback is that the link between proteins cannot translated into the link between disease and protein. It means that the interaction between two proteins is ubiquitous, not specific to a disease. Machine learning methods include Bayesian network method (Fu et al., 2017), Markov model method (Krejci et al., 2015; Guo et al., 2017), Random Forest method (Cheng et al., 2018b; Lv et al., 2019a,b; Xu et al., 2019) and Support Vector Machine method (Cui et al., 2018; Chao et al., 2019; Dao et al., 2020; Zhang et al., 2020) etc. Barber et al. (2017) uses Simulated Annealing (SA) to select the proteins most relevant to AD and uses Random Forest (RF) to classify patients based on these proteins. The best model trained in serum can significantly predict disease status with AUC of 0.66. At the same time, training with serum data and testing by CSF data, the AUC is 0.77. We once used Gradient Descent (GD) and Logistic Regression (LR) to identify AD-related proteins (Zhao et al., 2019b). But the precision of LRGD is not very high. There are two kinds of other methods to identify diseases-related proteins: Classification and Clustering. Clustering method belongs to unsupervised learning, and there are no fixed rules when pruning, which results in a lower interpretability of the results. The classification method requires a large number of negative samples, but for biological problems, negative samples are usually very difficult to obtain (Cheng et al., 2018a).

Diagnosis of AD by protein in blood is undoubtedly an exciting task (Feng, 2019; Zhou et al., 2019). Expensive and time-consuming biological experiments result in poor repeatability of research, and some of the results are contradictory. Discovering more AD potential blood proteins can not only greatly shorten the experimental cycle, but also help people understand the pathogenesis of AD in a deeper level. In this paper, the identification of AD-related proteins is regarded as an optimization problem, which avoids the problem of lack negative dataset in classification problems. The results will be compared with the InBioMap PPI network which published on “Nature Method” and the LRGD which we published before.

## Methods

### Work Frame

**Figure 1:**
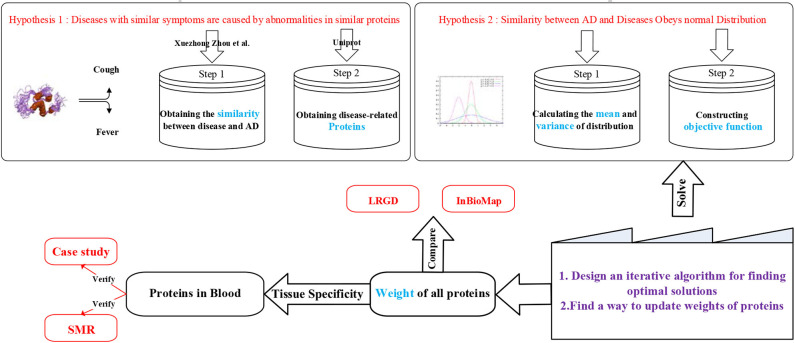
Work frame of IBDS.

As we can see in the Figure 1, we have two hypothesizes. Due to the hypothesis 1, we downloaded the result of Xuezhong Zhou et al.'s paper (Zhou et al., 2014). They calculated the similarity of different diseases by symptoms. In their paper, they said that the clinical manifestations of the disease are related to potential protein interactions. Since we got the similarity of diseases, we should obtain the known proteins for each disease. We obtained the data from UniProt (2016). Then, we could get diseases which are related to AD and their known corresponding proteins.

Here, we could find that some diseases have more than 100 kinds of known related proteins but some diseases only have few. Obviously, the proteins associated with each disease are not comprehensive enough to support the similarity of symptoms between diseases and AD. Therefore, we propose a second hypothesis. Obviously, the similarity of those diseases who have hundreds of related proteins is more dependable than those who have few proteins. Note that it does not mean that the similarity is undependable. It means that a part of the similarity of diseases with a small amount of known proteins is not expressed based on our existing knowledge, so their credibility declines. The next step is to estimate the mean and variance of each similarity. Here, we try to create an objective function which could map proteins to diseases' similarities. We will discuss this in the next section. We only have to know here is that every protein has its own weight and these weights could map to diseases' similarities and the aim of objective function is to make sure that the loss of mapping is minimum.

After constructing the objective function we should find a way to solve it. There are two steps. Firstly, we should design an iterative algorithm to find out the optimal solutions. Secondly, a reasonable method of updating protein weights needs to be designed.

After finding the optimal solutions, the weights of proteins could be known. We should sort the weight to find which proteins are most relevant to AD. In order to demonstrate the superiority of our method, we compare it with LRGD which we published before and InBioMap which published on “Nature Method.”

Then observe whether those proteins with significant weight are expressed in the blood. To verify these proteins, we did case studies and applied SMR to discuss them in gene level and biological experiments. SMR is a method to integrate GWAS and eQTL data together to find traits-related SNPs.

### Data Collection

Firstly, the similarity of diseases based on symptoms was downloaded from Zhou's et al. (2014) paper on the nature communications.

167 diseases have similarity with AD.

**Figure 2:**
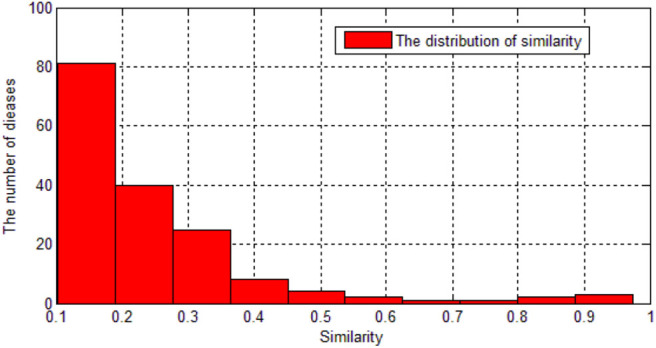
The distribution of similarity.

As we can see in the Figure 2, most of the diseases' similarity with AD are < 0.3. Therefore, we set 0.3 as a threshold. Then, there are 41 diseases remaining.

Next, the proteins which are related to the 41 diseases are downloaded from Uniprot. Among these diseases, we excluded Williams Syndrome because it is caused by gene deletion and it is related to more than 1,300 proteins in Uniprot. We also excluded Psychomotor Agitation and Ischemic Attack Transient because no related proteins could be found. Then we totally got 38 diseases which are related to AD and we got their known related proteins.

As we can see in the Table 1, we consider the similarity between AD and itself is 1. There is a large difference in the number of disease-related proteins we currently know. For example, 2 known proteins are related to Cadasil whereas Dementia has 123. Therefore, we cannot treat the similarity obtained as an exact number because there are some unknown proteins associated with these diseases and may be associated with AD and these unknown proteins may cause the similarity. Therefore, we assume that the similarity of each disease follows a normal distribution, and their similarity is the mean of the distribution. It can be denoted as *D*ise*ase*~*N*(*similarity*, σ^2^).

**Table 1:** Statistics of AD-related diseases.

**Disease**	**Similarity**	**Proteins**
Dementia	0.97	123
Huntington disease	0.36	43
Brain diseases	0.35	189
Bipolar disorder	0.40	52
Neurotoxicity syndromes	0.33	17
Glioblastoma	0.34	138
Encephalitis, herpes simplex	0.42	9
Multiple sclerosis, chronic progressive	0.39	12
Brain injuries	0.48	5
Glioma	0.32	122
Psychotic disorders	0.30	17
Cocaine-related disorders	0.32	6
Hashimoto disease	0.35	145
Substance-related disorders	0.37	9
Memory disorders	0.84	26
Dementia, vascular	0.90	17
Neurodegenerative diseases	0.44	36
Cerebral amyloid angiopathy	0.36	7
Herpes simplex	0.30	91
Schizophrenia	0.46	118
Brain ischemia	0.36	57
Cadasil	0.33	2
Depressive disorder	0.34	7
Cognition disorders	0.89	8
Epilepsy, temporal lobe	0.63	25
Alcoholism	0.44	7
Amphetamine-related disorders	0.75	5
Genetic predisposition to disease	0.32	52
Mood disorders	0.51	19
Nerve degeneration	0.58	66
Brain neoplasms	0.31	55
Hyperhomocysteinemia	0.42	5
Frontotemporal dementia	0.31	31
Periventricular nodular heterotopia	0.34	7
Frontotemporal lobar degeneration	0.52	7
Depressive disorder, major	0.56	5
Lewy body disease	0.35	22
Atrophy	0.82	342
Alzheimer's Disease	1	184

If we sum all the number of disease-related proteins together, the total number of proteins is 2,088. Actually, there are only 1,481 kinds of proteins. Therefore, some proteins are related to more than one disease. This also confirms our hypothesis: similar diseases share similar proteins.

### Method

#### Obtain the Variance of Diseases' Similarities

The uncertainty of diseases' similarities is caused by the unknown related proteins. Here, we used w to denote the probability that protein is related to disease. For each disease, the known related proteins are 1, then the unknown related proteins would be w_i_. Standard Error (SE) emphasizes the credibility of the mean. Here the mean is the diseases' similarity. The SE could be calculated as following:

(1) $$\begin{array}{l} \sigma \text{=}\sqrt{\frac{\bar{X}\left(1-\bar{X}\right)}{N}} \end{array}$$

N would be the number of proteins which is 1,481. $\bar{X}$ would be the mean of unknown proteins' probability.

So the variance of each disease's similarity could be calculated as:

(2) $$\begin{array}{l} \sigma^{2}\text{=(}\frac{\text{w(1-P)}}{1481}(1-\frac{\text{w(1-P)}}{1481}))/1481 \end{array}$$

w denotes the probability of each protein. P is the vector of relationship between protein and disease. We used one-hot encoding method to obtain P. For example, Brain Injuries is related to 5 proteins, then there are five 1 in P and other proteins are represented by w. It would be The position of 1 depends on the position of the protein associated with Brain Injuries in all 1,481 proteins.

In this way, diseases with more known proteins have a smaller variance than those with fewer known proteins.

#### Obtain Objective Function

Generally, the objective function of regression is as following,

(3) $$\begin{array}{l} L\text{=}\overset{n}{\underset{i=1}{\sum }}{\left(Y_{i}-{\overset{⌢}{Y}}_{i}\right)}^{2} \end{array}$$

*Y*_*i*_ is the true similarity, ${\overset{⌢}{Y}}_{i}$ is the similarity of our estimation. n is the number of related diseases.

Since *Y*_*i*_ is no longer a number but a distribution now, the objective function should be changed. Firstly, we should transform each Y into Standard Normal Distribution. Therefore, for each disease, Y should be:

(4) $$\begin{array}{l} {Y^{\prime }}_{i}\text{=(}Y_{i}\text{-}\mu_{i}\text{)/}\sigma_{i} \end{array}$$

μ_*i*_ denotes the similarity between i^th^ disease and AD, σ_*i*_ would be obtained by formula (2). Then the objective function should be as following:

(5) $$\begin{array}{l} L\text{=}\overset{39}{\underset{i=1}{\sum }}\overset{{\overset{⌢}{Y}}_{i}}{\underset{0}{\int }}{Y_{\text{i}}}^{\prime }dY \end{array}$$

So our goal is to find out the w which can minimize L.

#### Design an Iterative Algorithm for Finding Optimal Solution

Step 1. Set initial value

We totally got 184 known AD-related proteins, we assigned weights for these 184 proteins from 0.1 to 0.9. For example, if we assigned 0.1 to these 184 proteins, each protein's weight would be 0.1/184 = 0.000543. Then the rest proteins' weight would be 0.9/(1481–184) = 0.00069. If we got the similar results from different initial values, the result should be more reliable.

Step 2. Iteration rule

Since we have obtained some known AD-related proteins, the initial value of w should be very close to the final result. We do not want the final result to fall into local optimum, so in the iterative process, if the value of L is greater than the value of the previous iteration, the result is accepted with a variable probability.

In the initial iterations, the results are instability, so the probability of accepting the poorer new solutions is higher. As the number of iterations increases, this probability becomes smaller and smaller.

Step 3. Update weights

As we know, only few of these 1,481 proteins are related to AD. Then, we should decrease the weight of unrelated proteins and increase the weight of related proteins.

Since the number of features (protein) is far more than the number of samples (diseases), so it is reasonable to use the linear regression methods. Using Minimum angle regression (MAR) and Elastic Network (EN), the related proteins could be selected. For MAR, the unrelated proteins' regression coefficient would be 0. For EN, the unrelated proteins' regression coefficient would be quite low.

Both methods could find the related proteins, so they could help us update the weights of proteins. Since MAR only selects very few of proteins each time, we use EN to update weights four times, then use MAR once.

Note that the changes of weights will cause the changes in variance. Therefore, the objective function changes in every iteration. It is the reason that we do not skip the process of iteration and use MAR and EN only.

Since MAR and EN could found the related proteins, the weight of selected proteins will increase by a tenth of the minimum weight. To keep the sum of weights to be 1, unselected proteins will be subtracted some weights.

**Figure 3:**
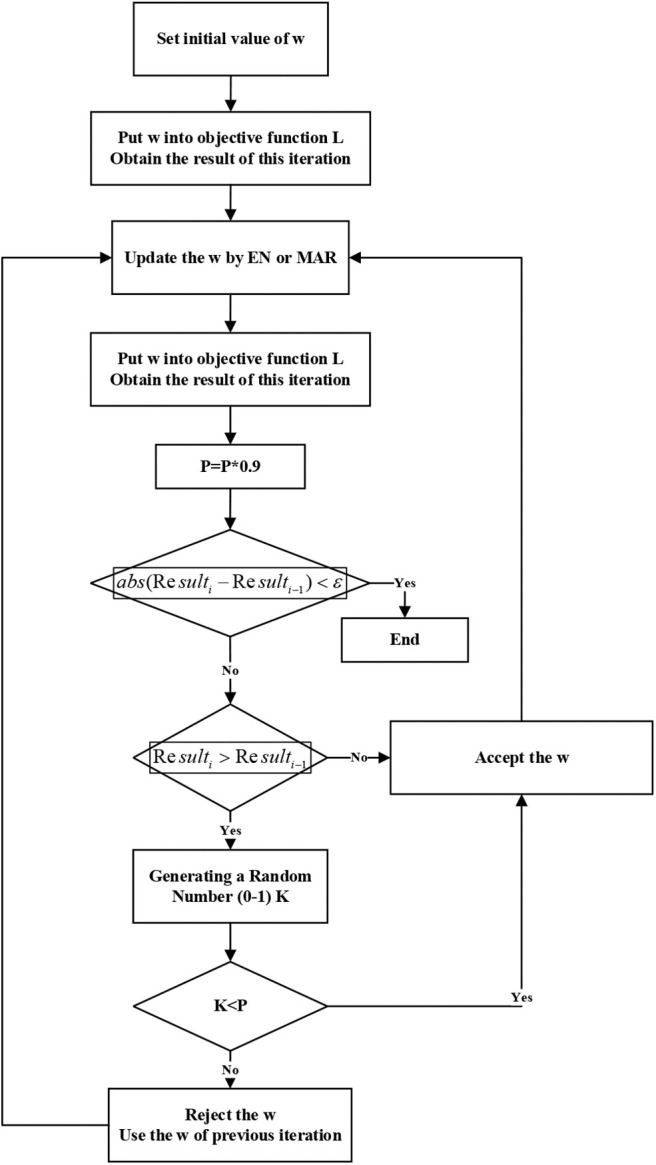
Work flow of finding optimal solution.

We will iterate until the value of the objective function converges. The work flow is shown in Figure 3.

## Results

### Result of Different Initial Values

Since the sum of initial value is assigned from 0.1 to 0.9, different initial values would cause the different results.

**Figure 4:**
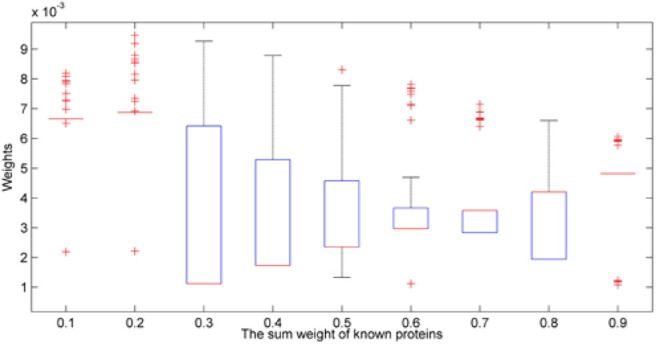
Box diagrams of result with different initial values.

We excluded the weights which are <0.001 and draw the Figure 4. As we can see in Figure 4, if we chose the extreme value like 0.1, 0.2, or 0.9, most protein weights are concentrated on a certain value, so there is no box in the box diagram but a line. The results of 0.6 and 0.7 are similar.

**Figure 5:**
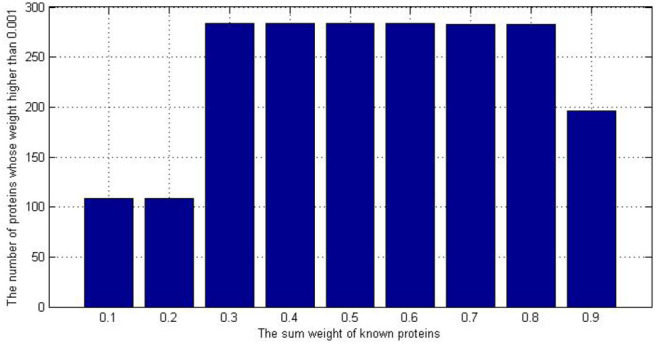
The number of potential AD-related protein with different initial values.

We also want to know how many proteins we have found to be related to AD. In Figure 5, we set a threshold as 0.001, if the weight of protein is higher than 0.001, we would consider it as a potential candidate protein which is related to AD.

As we can see in the Figure 5, if we chose the initial value 0.1 or 0.2, the number of potential AD-related would be 109. It is less than the 184 in the original dataset. Because the initial value did not make good use of our known prior information, that is, 184 known proteins are not distinguished significantly from other proteins, the iteration falls into local optimum. However, if we set the initial value as 0.9, We're too biased toward 184 known proteins so other unknown proteins are less likely to gain weight.

Therefore, we consider the most suitable initial value is 0.6. If the sum of known proteins' weight is 0.6, they are significant different from others and others unknown proteins got a chance to update their weights. We also summarized the results of all the initial value. We put all the results (9^*^1481) together and called it as ‘IBDS_summary initial value'. The AUC of it was 0.9107. Then, we try to compare our method with InBioMap and the method we published before (LRGD). InBioMap can find related proteins based on the input proteins and construct a protein network. We input the 184 kinds of known proteins to it and it uses the interaction between proteins to build a network. Then we used Random Walk (RW) which is a routine method to traverse the network. Then we could obtain the AD-related proteins by InBioMap. The method we published before is used to identify AD-related proteins too, so here we use the result of that paper directly.

**Figure 6:**
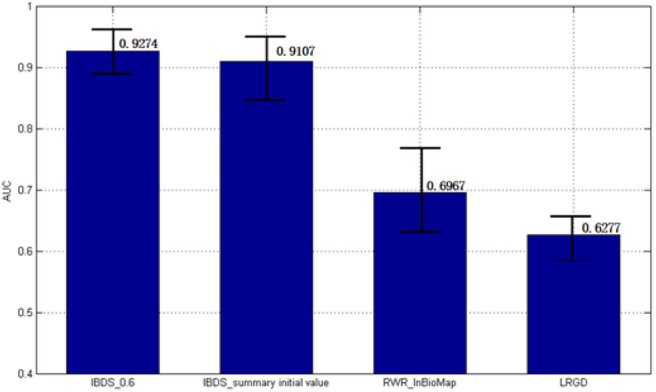
The AUC results of different methods.

Figure 6 shows AUC results of these four methods. IBDS performed best among these methods. In addition, the standard error of it was also the smallest, which means IBDS is stable.

Here, we want to analysis why our method performs better than these two methods. InBioMap only considers the interactions between proteins to build the network. If a protein is known to be associated with AD, although the more frequently it interacts with another protein, the more likely the protein is to be associated with AD, the interaction between the two proteins may not be related to AD. Therefore, only using the information between proteins would cause the bias. However, although LRGD considers the similarity between diseases, the objective function is not necessarily a convex function, so solving with the gradient descent method is risky. In addition, another problem is that we got the similarity of diseases by several methods, but all the methods are based on the genes' interaction. Since proteins cause the similarity of diseases due to the symptoms, we should calculate the similarity of diseases based on the similar symptoms. Therefore, in this paper, we chose the result of Zhou et al. which is obtained based on symptoms.

### Novel Proteins Expressed in Blood and Case Study

We totally obtained 284 proteins which are related to AD. Among them, there are 100 novel proteins. We obtained the related information about these 100 novel proteins from Uniprot. Since genes have tissue specificity (Zhao et al., 2020), we need to obtain proteins which mainly express in blood.

**Figure 7:**
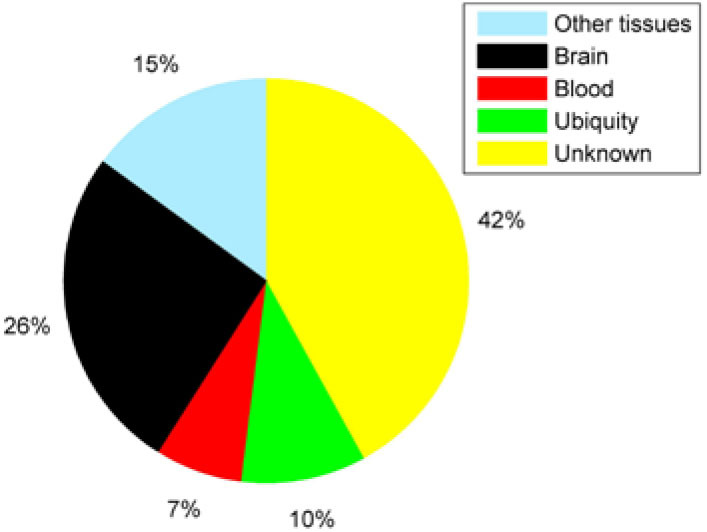
The distribution of novel proteins' tissue specificity.

As we can see in the Figure 7, 42% of proteins have not found tissue specificity at the mRNA level. Most of the remaining proteins have specificity expression in the brain. It is worth noting that there are 7 proteins that are specifically expressed in the blood.

Table 2 gives the name of these 7 proteins which have specificity expression in blood.

**Table 2:** Novel proteins specifically expressed in blood.

**ID**	**Name**	**Gene**
P31645	Sodium-dependent serotonin transporter	SLC6A4
P02751	Fibronectin	FN1
Q96P31	Fc receptor-like protein 3	FCRL3
Q99719	Septin-5	SEPT5
P17861	X-box-binding protein 1	XBP1
P00734	Prothrombin	F2
P01008	Antithrombin-III	SERPINC1

P31645 has some cognitive functions, including memory and learning. Regulation of serotonin at synapses is considered to be the main role of several antidepressant drugs. Therefore, the decline in P31645 expression levels is very likely to be a sign of AD. It has been reported that hippocampal P31645 sites are preserved or upregulated in physically aggressive AD patients relative to controls (Liu et al., 2018).

P02751 binds to the surface of lymphocyte, which improves the immune system and hinders the aging of the body. The absence of P02751 can cause defects in the mesoderm, nerve tubes and blood vessels. Lepelletier et al. (2017) have found that P02751expression was increased in subclinical AD and AD patients when compared to controls, in frontal and temporal cortex.

Q96P31 promotes TLR9-induced B-cell proliferation, activation and survival but inhibits antibody production and suppresses plasma cell differentiation. It is difficult to analyze the potential relationship between Q96P31 and AD from the existing literature.

Q99719 has been reported (Chang et al., 2015) that there is significant expression differences in hippocampus compared with motor cortex in AD and non-AD patients.

P17861 plays a role in regulating synaptic plasticity and memory function. Chang et al. (2015) reported that P17861 produces substantial protective effects on crucial neuronal circuitry involved in memory function.

In the recent paper published in Science Advances, P00734 has been considered as the most important feature to be a potential marker of early Aβ deposition. Chen et al. (2019) found that there is significate difference in serum P01008 between wild-type and AD mice.

In addition, we also did case studies on some other proteins which do not specifically express in blood. Q9NZC2 is encoded by TREM2, which has been reported to be associated with many neurodegenerative diseases such as Behavioral variant of frontotemporal dementia, Early-onset autosomal dominant Alzheimer disease (Jin et al., 2014), Progressive non-fluent aphasia, Semantic dementia. Nagar and Al-Mubaid (2008) found that P49810 is associated with AD based on gene ontology annotations. P49810 is coded by PSEN2 and expresses in many tissues such as heart, brain, placenta, liver, skeletal muscle, and kidney.

### Proof From SMR

SMR is a method which can integrate GWAS and eQTL data to identify the gene whose expression levels are associated with a complex trait because of pleiotropy. Zhu et al. (2016) purposed this method to identify several genes which are related to 5 complex traits. Since the paper was published, many researchers used SMR to identify diseases-related SNPs. This method can treat SNP as a tool variable to study the causal relationship between gene expression and disease. We also used this method to test AD-related genes before (Zhao et al., 2019c).

Since proteins are encoded by genes, we try to use SMR to determine whether the genes corresponding to the seven proteins affect AD through pleiotropy. In this paper, we used two GWAS datasets and one eQTL dataset to identify whether these 7 genes' expression levels are associated with AD.

eQTL data is from Lloyd-Jones et al. (2017) study. They analyzed the mRNA levels for 36,778 transcript expression traits (probes) from 2,765 individuals. GWAS data are from Lambert et al. (2013) and Marioni et al. (2018). Lambert used 17,008 AD cases and 37,154 controls. Marioni obtained genetic data from 314,278 individuals.

We did SMR test on the two GWAS datasets, respectively. Table 3 shows the summary results, of which *P*-values are the minimum *P*-value of the corresponding genes. We found significant SNPs of two genes. They are FCRL3 and XBP1.

**Table 3:** The number of SNPs of seven genes in data set.

**Gene**	**eQTL**	**GWAS**	***P*-value**
SLC6A4	1	0	/
FN1	17	16	0.0021
FCRL3	1,568	709	0.0003
SEPT5	7	6	0.0241
XBP1	2,902	1,241	0.0009
F2	0	0	/
SERPINC1	411	347	0.02

**Figure 8:**
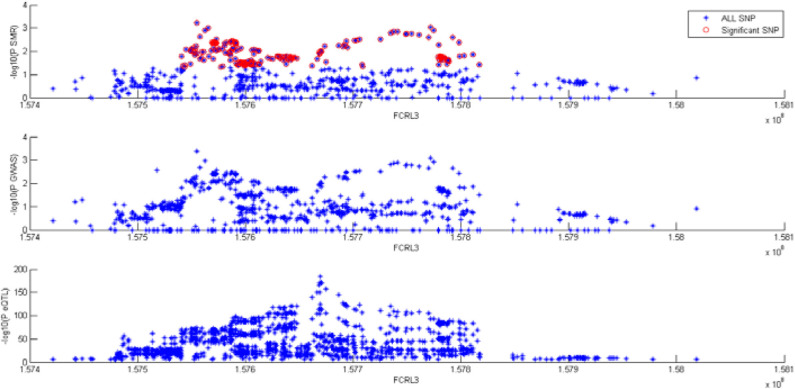
SMR tests for association between gene FCRL3 expression and complex traits.

Figure 8 shows the results at FCRL3 locus for AD. Top plot, all dots represent the *P*-value for SNPs from SMR test, red dots represent the *P*-value which is < 0.05. They are relative significant SNPs with a total of 474. The middle picture shows the *p*-value of SNPs in GWAS. If the two GWAS datasets contain a certain SNP, we select the one with the smaller *p*-value. Bottom plot, the eQTL *P*-values of SNPs from the Lloyd-Jones study for the ILMN_1699599 probe tagging FCRL3.

For space reasons, the results of XBP1 are not shown here, but a total of 10 SNPs have passed the SMR test.

## Conclusions

With the prolongation of human life span, more and more people are suffering from AD which consumes the most social resources. However, AD can only be diagnosed by autopsy and brain biopsy which is harmful. Increasing researchers have focus on diagnosing AD by urine and blood. Discovering more potential AD-related proteins could help us find a low-cost way to diagnose AD through blood.

In this paper, we propose a new method for identifying AD-related proteins, which combines elastic network with minimum-angle regression and finds the optimal solution by iteration. Different from the methods of protein network, we get the similarity of diseases according to the symptoms of diseases. Different diseases have different distribution of similarities. We get the variance of similarity distribution and construct a new objective function.

To verify the effectiveness of our method, we compared our method with two methods (LRGD and InBioMap) and did case studies. InBioMap is based on PPI network and LRGD is method we purposed before. The result shows our method IBDS (AUC = 0.9274) is better than LRGD (AUC = 0.6277) and RWR-InBioMap (AUC = 0.6967). In addition, we found 100 novel AD-related proteins and 7 of them have tissue specificity in blood. More importantly, some studies have confirmed that some of these novel proteins have differences in expression between AD patients and normal people.

## Data Availability Statement

Publicly available datasets were analyzed in this study. This data can be found here: https://static-content.springer.com/esm/art%3A10.1038%2Fncomms5212/MediaObjects/41467_2014_BFncomms5212_MOESM1046_ESM.txt.

## Author Contributions

TZh wrote the paper and did the experiments. YH provided ideas of this work. TZa revised this manuscript and guided how to do experiments. YW supervised this work. All authors contributed to the article and approved the submitted version.

## Conflict of Interest

The authors declare that the research was conducted in the absence of any commercial or financial relationships that could be construed as a potential conflict of interest.
